# (*S*)-2-Amino-1-(pyrrolidinium-2-ylmeth­yl)pyridinium dibromide

**DOI:** 10.1107/S1600536808001128

**Published:** 2008-01-18

**Authors:** Yifeng Wang, Shuai Zhang, Aibao Xia, Shuping Luo, Danqian Xu

**Affiliations:** a State Key Laboratory Breeding Base of Green Chemistry – Synthesis Technology, Zhejiang University of Technology, Hangzhou 310014, People’s Republic of China

## Abstract

In the title compound, C_10_H_17_N_3_^2+^·2Br^−^, the pyrrolidinium ring displays an envelope conformation, with the flap N atom lying 0.564 (6) Å from the mean plane of the remaining four C atoms. The attached methyl­ene C atom, which connects the pyrrolidinium ring and the 2-amino­pyridine group, is displaced from the plane of the four pyrrolidinium C atoms by 0.811 (8) Å in the same direction as the pyrrolidinium N atom. The amine N lies on the opposite side of this plane.

## Related literature

The synthesis of (*S*)-(+)-2-bromo­methyl­pyrrolidine hydro­bromide is described by Xu *et al.* (2006 ▶). For related literature, see: Ishii *et al.* (2004 ▶); Larson (1970 ▶).
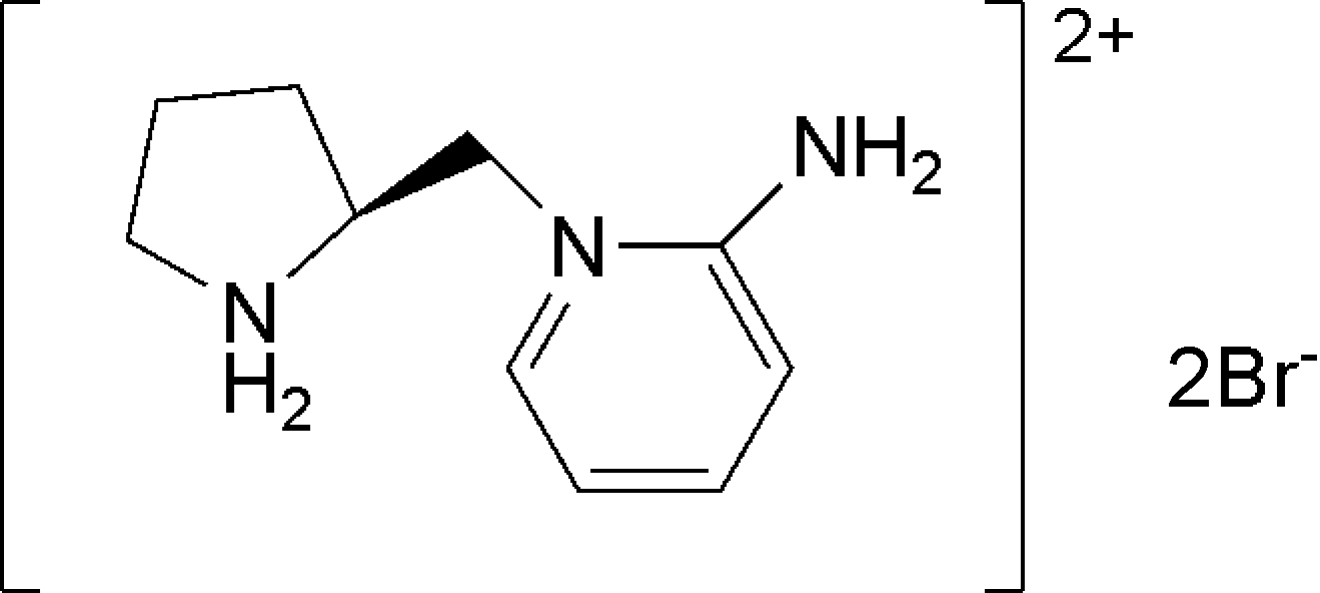


## Experimental

### 

#### Crystal data

C_10_H_17_N_3_^2+^·2Br^−^
*M* *_r_* = 339.07
Monoclinic, 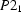

*a* = 10.5509 (5) Å
*b* = 6.1755 (3) Å
*c* = 10.8474 (6) Å
β = 107.4830 (14)°
*V* = 674.14 (6) Å^3^
*Z* = 2
Mo *K*α radiation
μ = 6.01 mm^−1^
*T* = 296 (1) K
0.37 × 0.32 × 0.13 mm

#### Data collection

Rigaku R-AXIS RAPID diffractometer
Absorption correction: multi-scan (*ABSCOR*; Higashi, 1995 ▶) *T*_min_ = 0.110, *T*_max_ = 0.458
6601 measured reflections
2681 independent reflections
1943 reflections with *F* ^2^ > 2σ(*F* ^2^)
*R*_int_ = 0.052

#### Refinement

*R*[*F* ^2^ > 2σ(*F* ^2^)] = 0.034
*wR*(*F* ^2^) = 0.094
*S* = 1.01
2681 reflections
138 parameters
H-atom parameters constrained
Δρ_max_ = 0.41 e Å^−3^
Δρ_min_ = −0.61 e Å^−3^
Absolute structure: Flack (1983 ▶), with 1013 Friedel pairs
Flack parameter: 0.002 (5)

### 

Data collection: *PROCESS-AUTO* (Rigaku, 1998 ▶); cell refinement: *PROCESS-AUTO*; data reduction: *CrystalStructure* (Rigaku/MSC, 2004 ▶); program(s) used to solve structure: *SHELXS97* (Sheldrick, 2008 ▶); program(s) used to refine structure: *CRYSTALS* (Betteridge *et al.*, 2003 ▶); molecular graphics: *CrystalStructure*; software used to prepare material for publication: *CrystalStructure*.

## Supplementary Material

Crystal structure: contains datablocks global, I. DOI: 10.1107/S1600536808001128/cs2067sup1.cif

Structure factors: contains datablocks I. DOI: 10.1107/S1600536808001128/cs2067Isup2.hkl

Additional supplementary materials:  crystallographic information; 3D view; checkCIF report

## Figures and Tables

**Fig. 1.**
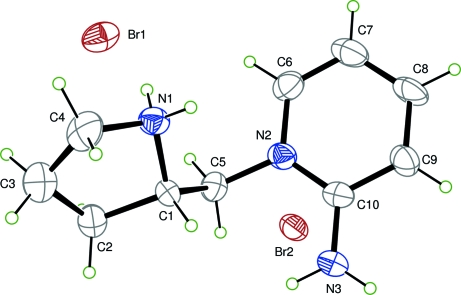
The asymmetric unit of the crystal structure of the title compound with the atomic labeling scheme. Displacement ellipsoids are drawn at the 40% probability level.

## supplementary crystallographic information

### Comment

Proline and its derivatives have been extensively studied due to their abilities to catalyze a wide range of reactions as organocatalysts in recent years (Ishii *et al.*, 2004; Xu *et al.*, 2006). The title compound, which could be readily synthesized from commercially available *L*-proline and 2-aminopyridine, can act as organocatalyst in the Michael addition of ketones to nitrostyrenes. These reactions afford the desired Michael adducts in good yields and moderate enantioselectivities. The title salt (*S*)-2-amino-1-(pyrrolidinium-2-ylmethyl)-pyridinium dibromide crystal structure (Fig. 1) is built of pyrrolidinium cations and bromide anions. The pyrrolidinium ring displays a fair half-chair conformation, with the flap atom N1 lying 0.564 (6) Å from the mean plane of C1/C2/C3/C4. The methylene C5 atom, which connects the pyrrolidinium ring and the 2-aminopyridine group, is displaced from the plane of four pyrrolidinium carbons by 0.811 (8) Å in the same direction as the N1 atom. The atom N3 of the amino group of pyrrolidinium and the atom N1 are on the opposite sides of the mean plane of C1/C2/C3/C4.

### Experimental

The title compound was synthesized by treating 2-aminopyridine (0.94 g,10 mmol) with (*S*)-(+)-2-bromomethylpyrrolidine hydrobromide (2.50 g,10 mmol) in MeCN (30 ml) under stirring at 353 K for 24 h (yield 92%). The compound (*S*)-(+)-2-bromomethylpyrrolidine hydrobromide was obtained from commercially available *L*-proline by reduction with NaBH_4_ and subsequent bromination with PBr_3_ (Xu *et al.*, 2006). Suitable crystals of the title compound were obtained by slow evaporation of an ethanol solution at room temperature.

### Refinement

H atoms were placed in calculated position with N—H=0.86 Å, C—H=0.98 Å(*sp*), C—H=0.97 Å(sp2), C—H=0.93 Å(aromatic). All H atoms included in the final cycles of refinement as riding mode, with *U*_iso_(H)=1.2*U*_eq_ of the carrier atoms.

### Crystal data

**Table:**

C_10_H_17_N_3_^2+^·2Br^–^	*F*_000_ = 336.00
*M**_r_* = 339.07	*D*_x_ = 1.670 Mg m^−^^3^
Monoclinic, *P*2_1_	Mo *K*α radiation λ = 0.71075 Å
Hall symbol: P 2yb	Cell parameters from 5483 reflections
*a* = 10.5509 (5) Å	θ = 3.2–27.5º
*b* = 6.1755 (3) Å	µ = 6.01 mm^−^^1^
*c* = 10.8474 (6) Å	*T* = 296 (1) K
β = 107.4830 (14)º	Platelet, colorless
*V* = 674.14 (6) Å^3^	0.37 × 0.32 × 0.13 mm
*Z* = 2	

### Data collection

**Table:**

Rigaku R-AXIS RAPID diffractometer	1943 reflections with *F*^2^ > 2σ(*F*^2^)
Detector resolution: 10.00 pixels mm^-1^	*R*_int_ = 0.052
ω scans	θ_max_ = 27.5º
Absorption correction: multi-scan(ABSCOR; Higashi, 1995)	*h* = −13→13
*T*_min_ = 0.110, *T*_max_ = 0.458	*k* = −7→8
6601 measured reflections	*l* = −14→14
2681 independent reflections	

### Refinement

**Table:**

Refinement on *F*^2^	(Δ/σ)_max_ < 0.001
*R*[*F*^2^ > 2σ(*F*^2^)] = 0.034	Δρ_max_ = 0.41 e Å^−^^3^
*wR*(*F*^2^) = 0.094	Δρ_min_ = −0.61 e Å^−^^3^
*S* = 1.01	Extinction correction: Larson (1970), equation 22
2681 reflections	Extinction coefficient: 48 (6)
138 parameters	Absolute structure: Flack (1983), 1013 Friedel pairs
H-atom parameters constrained	Flack parameter: 0.002 (5)
*w* = 1/[0.9800σ(*F*_o_^2^)]/(4*F*_o_^2^)	

### Fractional atomic coordinates and isotropic or equivalent isotropic displacement parameters (Å^2^)

**Table:**

	*x*	*y*	*z*	*U*_iso_*/*U*_eq_	
Br1	0.12426 (6)	0.7373 (2)	0.07481 (6)	0.0544 (2)	
Br2	0.36075 (6)	0.4571 (2)	0.62553 (6)	0.0481 (2)	
N1	0.2124 (4)	0.2325 (8)	0.0528 (4)	0.0394 (13)	
N2	0.2014 (4)	0.1711 (7)	0.3238 (4)	0.0383 (14)	
N3	0.3461 (4)	−0.1049 (8)	0.4252 (5)	0.0504 (17)	
C1	0.3320 (5)	0.2156 (10)	0.1669 (5)	0.0373 (16)	
C2	0.4356 (6)	0.3574 (10)	0.1293 (7)	0.050 (2)	
C3	0.3902 (8)	0.3654 (13)	−0.0157 (8)	0.072 (3)	
C4	0.2652 (7)	0.2334 (12)	−0.0600 (6)	0.058 (2)	
C5	0.3056 (6)	0.2905 (8)	0.2915 (6)	0.0396 (19)	
C6	0.0744 (6)	0.2588 (11)	0.2846 (6)	0.0473 (19)	
C7	−0.0286 (6)	0.1613 (11)	0.3117 (7)	0.058 (2)	
C8	−0.0044 (5)	−0.0355 (14)	0.3828 (6)	0.054 (2)	
C9	0.1193 (6)	−0.1204 (10)	0.4205 (6)	0.048 (2)	
C10	0.2260 (5)	−0.0171 (10)	0.3902 (5)	0.0399 (16)	
H5	0.3628	0.0650	0.1770	0.045*	
H6	0.0599	0.3881	0.2384	0.057*	
H7	−0.1133	0.2214	0.2845	0.070*	
H8	−0.0734	−0.1056	0.4033	0.065*	
H9	0.1347	−0.2491	0.4671	0.057*	
H21	0.4378	0.5019	0.1650	0.060*	
H22	0.5232	0.2927	0.1605	0.060*	
H31	0.3725	0.5138	−0.0450	0.087*	
H32	0.4578	0.3046	−0.0494	0.087*	
H41	0.2847	0.0872	−0.0817	0.070*	
H42	0.2022	0.2998	−0.1346	0.070*	
H51	0.3871	0.2743	0.3622	0.048*	
H52	0.2804	0.4420	0.2820	0.048*	
H111	0.1698	0.3502	0.0555	0.047*	
H112	0.1607	0.1234	0.0491	0.047*	
H301	0.4104	−0.0412	0.4065	0.061*	
H302	0.3597	−0.2257	0.4666	0.061*	

### Atomic displacement parameters (Å^2^)

**Table:**

	*U*^11^	*U*^22^	*U*^33^	*U*^12^	*U*^13^	*U*^23^
Br1	0.0568 (4)	0.0313 (3)	0.0617 (4)	0.0003 (3)	−0.0024 (3)	0.0000 (3)
Br2	0.0345 (3)	0.0652 (4)	0.0452 (4)	−0.0024 (3)	0.0130 (2)	0.0014 (3)
N1	0.035 (2)	0.038 (2)	0.044 (3)	0.003 (2)	0.011 (2)	0.000 (2)
N2	0.035 (2)	0.047 (3)	0.036 (3)	0.005 (2)	0.014 (2)	0.000 (2)
N3	0.031 (2)	0.060 (3)	0.063 (4)	0.008 (2)	0.017 (2)	0.015 (2)
C1	0.030 (2)	0.042 (3)	0.043 (3)	0.001 (2)	0.015 (2)	0.008 (2)
C2	0.047 (4)	0.057 (4)	0.048 (4)	−0.004 (3)	0.017 (3)	0.007 (3)
C3	0.070 (5)	0.088 (6)	0.065 (6)	0.003 (4)	0.030 (4)	0.016 (4)
C4	0.075 (4)	0.071 (4)	0.032 (3)	0.012 (5)	0.022 (3)	−0.003 (3)
C5	0.044 (3)	0.040 (4)	0.035 (3)	−0.002 (2)	0.013 (3)	0.002 (2)
C6	0.050 (3)	0.053 (3)	0.039 (3)	0.009 (3)	0.013 (3)	0.006 (3)
C7	0.034 (3)	0.084 (5)	0.057 (4)	0.004 (3)	0.015 (3)	−0.004 (3)
C8	0.029 (2)	0.088 (5)	0.052 (4)	0.006 (4)	0.023 (2)	−0.001 (4)
C9	0.041 (3)	0.064 (5)	0.042 (4)	−0.004 (3)	0.018 (3)	0.004 (2)
C10	0.031 (2)	0.047 (3)	0.039 (3)	0.003 (3)	0.008 (2)	−0.003 (3)

### Geometric parameters (Å, °)

**Table:**

N1—C1	1.483 (6)	N3—H301	0.860
N1—C4	1.490 (9)	N3—H302	0.860
N2—C5	1.451 (8)	C1—H5	0.980
N2—C6	1.388 (8)	C2—H21	0.970
N2—C10	1.351 (7)	C2—H22	0.970
N3—C10	1.325 (7)	C3—H31	0.970
C1—C2	1.547 (9)	C3—H32	0.970
C1—C5	1.532 (9)	C4—H41	0.970
C2—C3	1.501 (9)	C4—H42	0.970
C3—C4	1.501 (9)	C5—H51	0.970
C6—C7	1.350 (9)	C5—H52	0.970
C7—C8	1.421 (9)	C6—H6	0.930
C8—C9	1.351 (9)	C7—H7	0.930
C9—C10	1.415 (9)	C8—H8	0.930
N1—H111	0.860	C9—H9	0.930
N1—H112	0.860		
			
C1—N1—C4	104.6 (4)	C1—C2—H21	110.5
C5—N2—C6	117.3 (4)	C1—C2—H22	110.5
C5—N2—C10	121.8 (4)	C3—C2—H21	110.5
C6—N2—C10	120.8 (5)	C3—C2—H22	110.5
N1—C1—C2	103.4 (4)	H21—C2—H22	109.5
N1—C1—C5	112.4 (4)	C2—C3—H31	110.1
C2—C1—C5	113.1 (4)	C2—C3—H32	110.1
C1—C2—C3	105.5 (5)	C4—C3—H31	110.1
C2—C3—C4	106.9 (7)	C4—C3—H32	110.1
N1—C4—C3	104.4 (5)	H31—C3—H32	109.5
N2—C5—C1	114.3 (4)	N1—C4—H41	110.7
N2—C6—C7	121.7 (6)	N1—C4—H42	110.7
C6—C7—C8	118.3 (6)	C3—C4—H41	110.7
C7—C8—C9	119.7 (6)	C3—C4—H42	110.7
C8—C9—C10	121.2 (6)	H41—C4—H42	109.5
N2—C10—N3	121.3 (5)	N2—C5—H51	108.3
N2—C10—C9	118.2 (5)	N2—C5—H52	108.3
N3—C10—C9	120.5 (5)	C1—C5—H51	108.3
C1—N1—H111	110.7	C1—C5—H52	108.3
C1—N1—H112	110.7	H51—C5—H52	109.5
C4—N1—H111	110.7	N2—C6—H6	119.2
C4—N1—H112	110.7	C7—C6—H6	119.2
H111—N1—H112	109.5	C6—C7—H7	120.8
C10—N3—H301	120.0	C8—C7—H7	120.8
C10—N3—H302	120.0	C7—C8—H8	120.1
H301—N3—H302	120.0	C9—C8—H8	120.1
N1—C1—H5	109.3	C8—C9—H9	119.4
C2—C1—H5	109.3	C10—C9—H9	119.4
C5—C1—H5	109.3		
			
C1—N1—C4—C3	−38.1 (6)	N1—C1—C2—C3	−23.1 (6)
C4—N1—C1—C2	37.7 (6)	N1—C1—C5—N2	58.8 (6)
C4—N1—C1—C5	159.9 (5)	C2—C1—C5—N2	175.4 (4)
C5—N2—C6—C7	−179.0 (6)	C5—C1—C2—C3	−144.9 (5)
C6—N2—C5—C1	−95.2 (6)	C1—C2—C3—C4	0.0 (6)
C5—N2—C10—N3	−2.4 (8)	C2—C3—C4—N1	23.0 (7)
C5—N2—C10—C9	178.4 (5)	N2—C6—C7—C8	0.3 (8)
C10—N2—C5—C1	85.4 (6)	C6—C7—C8—C9	−0.4 (9)
C6—N2—C10—N3	178.2 (5)	C7—C8—C9—C10	−0.1 (8)
C6—N2—C10—C9	−0.9 (8)	C8—C9—C10—N2	0.8 (9)
C10—N2—C6—C7	0.4 (8)	C8—C9—C10—N3	−178.4 (6)
